# Discrete Emotion Networks Across the Lifespan: Implications for Well-Being

**DOI:** 10.1093/geroni/igaa057.2141

**Published:** 2020-12-16

**Authors:** Meaghan Barlow, Iris Mauss

**Affiliations:** 1 University of California, Berkeley, Berkeley, California, United States; 2 Univerity of California, Berkeley, Berkeley, California, United States

## Abstract

Research examining the age-related trajectories and consequences of emotional complexity has largely lumped emotions into broad categories. The present study utilized network analyses to quantify the co-occurrence of discrete emotions and their associations with well-being across the lifespan in a sample of 156 females (aged 23-79). Participants completed assessments of 8 emotions (i.e., sad, angry, anxious, lonely, happy, excited, proud, and calm) for 16 days, and completed measures of psychological and physical well-being at a 4-month follow-up. While certain emotions were found to co-occur at similar rates across the lifespan (e.g. sad-anxious), other emotion pairs become more (e.g. sad-calm) or less (e.g. sad-angry) frequent with age. Additionally, specific emotion pairs were differentially associated with indicators of well-being across the lifespan, while controlling for mean levels of these emotions. These findings point to the importance of considering the co-occurrence of distinct emotions and potential pathways towards successful aging.

